# Comprehensive analysis of the long noncoding RNA-associated competitive endogenous RNA network in the osteogenic differentiation of periodontal ligament stem cells

**DOI:** 10.1186/s12864-021-08243-4

**Published:** 2022-01-03

**Authors:** Lingzhi Lai, Zhaodan Wang, Yihong Ge, Wei Qiu, Buling Wu, Fuchun Fang, Huiyong Xu, Zhao Chen

**Affiliations:** 1 Department of Stomatology of Maoming People’s Hospital, Maoming, 525000 China; 2 Department of Stomatology of Nanfang Hospital, Southern Medical University, Guangzhou, Guangdong 510515 People’s Republic of China; 3 Shenzhen Stomatology Hospital (Pingshan), Southern Medical University, 143 Dongzong Road, Pingshan District, Shenzhen, 518118 China

**Keywords:** Competing endogenous RNA, Long noncoding RNA, MicroRNA, osteogenic differentiation, Periodontal ligament stem cells

## Abstract

**Backgroud:**

The mechanism implicated in the osteogenesis of human periodontal ligament stem cells (PDLSCs) has been investigated for years. Previous genomics data analyses showed that long noncoding RNA (lncRNA), microRNA (miRNA) and messenger RNA (mRNA) have significant expression differences between induced and control human PDLSCs. Competing for endogenous RNAs (ceRNA), as a widely studied mechanism in regenerative medicine, while rarely reported in periodontal regeneration. The key lncRNAs and their ceRNA network might provide new insights into molecular therapies of periodontal regeneration based on PDLSCs.

**Results:**

Two networks reflecting the relationships among differentially expressed RNAs were constructed. One ceRNA network was composed of 6 upregulated lncRNAs, 280 upregulated mRNAs, and 18 downregulated miRNAs. The other network contained 33 downregulated lncRNAs, 73 downregulated mRNAs, and 5 upregulated miRNAs. Functional analysis revealed that 38 GO terms and 8 pathways related with osteogenesis were enriched. Twenty-four osteogenesis-related gene-centred lncRNA-associated ceRNA networks were successfully constructed. Among these pathways, we highlighted MAPK and TGF-beta pathways that are closely related to osteogenesis. Subsequently, subnetworks potentially linking the GO:0001649 (osteoblast differentiation), MAPK and TGF-beta pathways were constructed. The qRT-PCR validation results were consistent with the microarray analysis.

**Conclusion:**

We construct a comprehensively identified lncRNA-associated ceRNA network might be involved in the osteogenesis of PDLSCs, which could provide insights into the regulatory mechanisms and treatment targets of periodontal regeneration.

**Supplementary Information:**

The online version contains supplementary material available at 10.1186/s12864-021-08243-4.

## Background

Periodontal ligament stem cells (PDLSCs), as the key cells that maintain the dynamic balance of periodontal tissue and repair defects, are considered to be important seed cells in periodontal tissue engineering [1, 2]. The differentiation of PDLSCs into osteoblasts is an important step in periodontal tissue engineering [3, 4]. Studies have explored the mechanisms of this process [5–7]. However, the precise molecular mechanisms remain unclear. Thus, there is an urgent need to elucidate the mechanism of osteogenic differentiation of PDLSCs and develop novel targets for periodontal repair and regeneration.

Noncoding RNA (ncRNA), accounting for 90% of the human transcriptome, has been revealed to play a pivotal role in various biological processes via interference with gene expression [8, 9]. Emerging evidence has shown that the dysregulated expression of ncRNAs is associated with numerous diseases [10, 11]. lncRNAs with a length of more than 200 nucleotides were initially regarded as “transcriptional noise” and nonfunctional [12]. However, recent studies have revealed that lncRNAs participate in genome organization and in life processes such as growth and development, proliferation, differentiation, apoptosis of cells and immune responses [13–15].

The osteogenic differentiation of PDLSCs has an effect on epigenetic regulation, subsequently causing changes in gene expression [16–18]. Our previous study found that 2171 lncRNAs and 3557 messenger RNAs (mRNAs) were significantly differentially expressed during the osteogenesis of PDLSCs. Our other research used a microarray to identify the microRNA (miRNA) pattern during the osteogenic differentiation of PDLSCs [19]. It was found that the miRNAs with significantly different expression might function in this process by regulating targets, including osteogenesis-related genes [20]. The two studies demonstrated that lncRNAs and miRNAs might play an essential role in the osteogenic differentiation processes of PDLSCs.

**Fig. 1:**
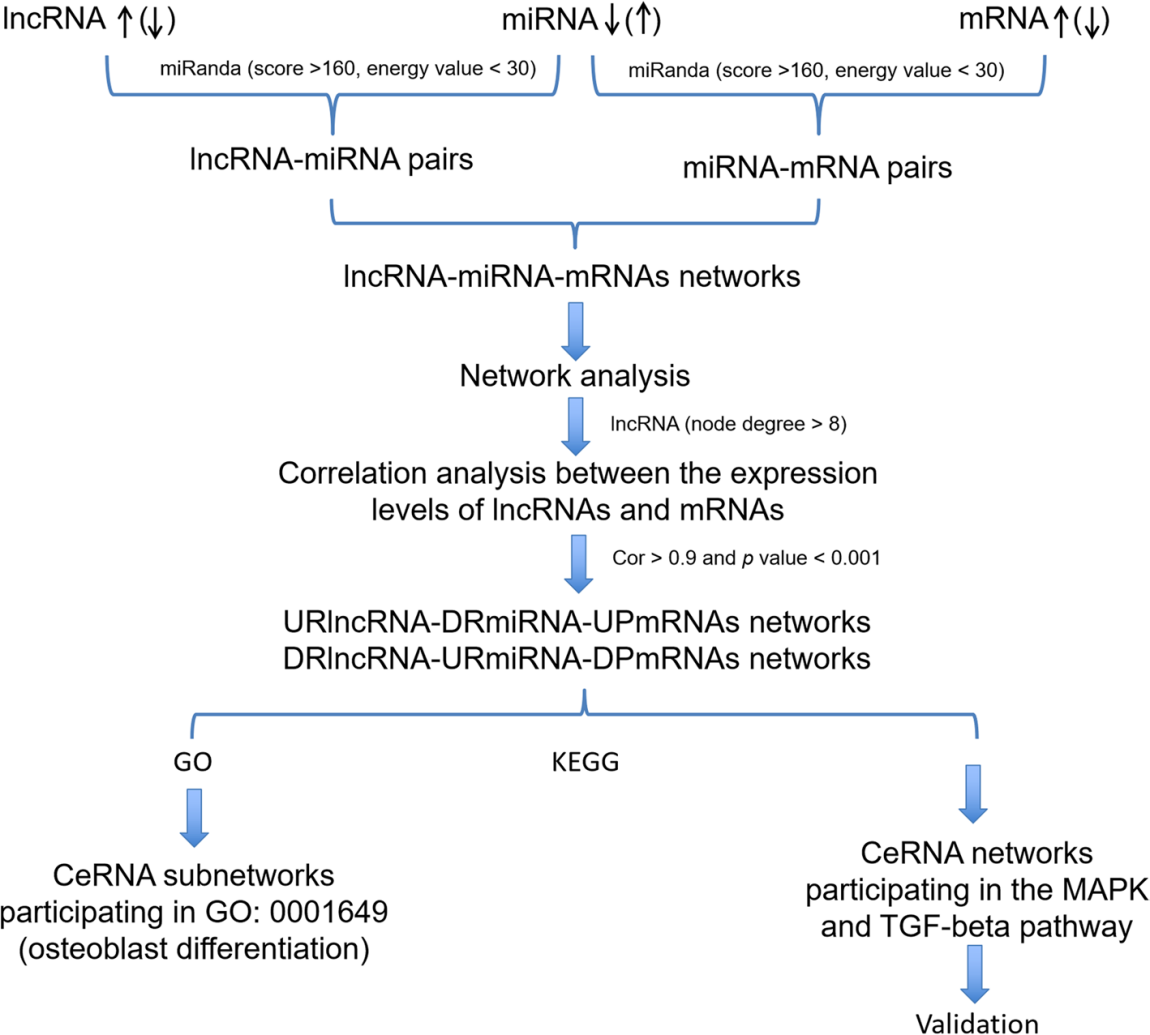
The reconstruction process of the lncRNA–miRNA–mRNA network. First, miRNA expression data were downloaded from two previous studies (GSE159507 and GSE159508). Second, differentially expressed mRNAs, lncRNAs, and miRNAs were screened. Third, target mRNAs of miRNAs were predicted using miRanda, and target lncRNAs of differentially expressed microRNAs were predicted using miRanda. Then, differentially expressed lncRNAs and mRNAs were merged with the target lncRNAs and mRNAs of differentially expressed miRNAs, respectively. The node degree of the selected lncRNA was more than 8. The coexpressed lncRNAs and mRNAs were selected (Pearson correlation coefficient > 0.90 and *P* < 0.001). Finally, the differentially expressed miRNAs, coexpressed lncRNAs and mRNAs were mapped into the interactions. lncRNA, long noncoding RNA; miRNA, microRNA; mRNA, messenger RNA

In 2014, Pandolfi *et al.* revealed a novel regulatory mechanism of competing endogenous RNAs (ceRNAs), in which RNA transcripts, including lncRNAs, pseudogenes, circular RNAs, *etc.,* with miRNA-binding sites could modulate miRNA-based regulation [21–23]. In 2018, Gu *et al.* screened lncRNAs and circRNAs differentially expressed during the osteogenic differentiation of PDLSCs by RNA sequencing, predicted miRNAs that might bind with them by bioinformatics analysis, and established a ceRNA network [6]. However, the expression and regulation of lncRNAs in the osteogenic differentiation of human PDLSCs are not fully understood. In our study, the same batch of samples was used to obtain differentially expressed lncRNAs, miRNAs and mRNAs through a microarray [19, 20], and these differentially expressed RNAs were used to construct a ceRNA network to obtain more reliable ceRNA data. Thus, our study attempted to identify a molecular interactive network of lncRNAs, miRNAs, and mRNAs (Fig. 1) using a variety of relevant databases. Further, validation of the key lncRNA–miRNA–mRNA axis was conducted. The key lncRNAs and their ceRNA network might provide new insights into molecular therapies for periodontal regeneration based on PDLSCs

## Materials and methods

### PDLSCs isolation, culture and osteogenic induction

The PDLSCs were collected from the third molars extracted from individuals. The standards of the collected teeth: (1) healthy, without oral or systemic diseases; (2) aged from 18 to 25 years; (3) containing both females and males. (4) non-pregnant and non-nursing. Subjects were recruited at Nanfang Hospital, Southern Medical University, Guangzhou, China. Informed consents were obtained from all donors, which can be seen in Table S4. The teeth were washed with phosphate-buffered saline (PBS) for several times to clean the blood. Then, the ligament tissues were separated from the middle 1/3 root surface of the teeth and cut into 1mm3 debris. After 20-minute-digestion with 3 mg/mL collagenase type I, the tissue debris were put into T25 flasks and maintained in DMEM complete medium under standard cell culture condition. For osteogenic induction, the PDLSCs were cultured in completed DMEM medium contained 100 nM dexamethasone, 10 mmol/L β-glycerophosphate, and 50 μg/mL ascorbic acid. The methods to verify the success of PDLSCs induction can be found in Supplementary Methods and Figure S1.

### Raw data

Human lncRNA, miRNA and mRNA expression data were downloaded from our two previous studies (GSE159507 and GSE159508). Two previous studies explored the lncRNA-miRNA-mRNA profile during the osteoblast differentiation of human PDLSCs.

### Screening the dysregulated lncRNAs, miRNAs and mRNAs

Two-class differential analysis was applied to determine the significant difference between dysregulated lncRNAs, miRNAs and mRNAs in osteogenic inductive PDLSCs compared with noninductive PDLSCs. The significantly dysregulated lncRNAs, miRNAs and mRNAs were recruited according to fold change > 2 and *P*-value < 0.05.

### Prediction of target lncRNAs and mRNAs of dysregulated miRNAs

The lncRNA targets of miRNAs were predicted using miRanda tools. A score >160 and energy value < 30 threshold were applied to predict the target lncRNAs in the prediction module. The target mRNAs of the selected miRNAs were predicted using the online miRNA reference database miRanda. Target genes were selected based on identification by this program. The specific binding sites were predicted by RNAhybrid.

### LncRNA-miRNA-mRNA regulatory networks

The lncRNA–miRNA–mRNA networks were established according to ceRNA theory [21–23]. First, miRanda was used to predict miRNA-bound mRNA and miRNA-bound lncRNA. Second, in a coexpressed competing triplet, both lncRNAs and mRNAs in the coexpression pattern were predicted and coexpressed negatively with the same miRNA [24]. Finally, the identified co-expressed competing triplets were used to construct a lncRNA–miRNA–mRNA network, which was visualized through Cytoscape software. Two networks were built according to the expression level of lncRNAs, miRNAs, and mRNAs. One network included lncRNAs with upregulated expression (URLs), miRNAs with downregulated expression (DRMis) and mRNAs with upregulated expression (URMs). The other contained lncRNAs with downregulated expression (DRLs), miRNAs with upregulated expression (URMis) and mRNAs with downregulated expression (DRMs).

Hub nodes play essential roles in regulating networks. Hence, the node degrees of the URL–DRM–URM and DRL–URM–DRM networks were calculated. A node with a degree exceeding 8 was identified as a hub node. LncRNAs with a node degree of more than 8 were extracted with their linked miRNAs and mRNAs, and Cytoscape software was applied to construct the lncRNA-miRNA-mRNA ceRNA networks.

### Functional enrichment analysis

Gene Ontology (GO) biological processes of mRNAs with upregulated and downregulated expression involved in the URL–DRM–URM and DRL–URM–DRM networks were analysed using the Database for Annotation, Visualization, and Integration Discovery (DAVID). Pathway analysis was applied according to the Kyoto Encyclopedia of Genes and Genomes (KEGG) databas e[25–27].

### qRT-PCR validation

Total RNAs were isolated from PDLSCs using EZ-press RNA Purification Kit (EZBioscience, USA), mRNAs were reverse-transcribed into cDNAs by the Color Reverse Transcription Kit (EZBioscience, Roseville, USA) and the cDNAs of miRNAs were acquired with the microRNA Reverse Transcription Kit (EZBioscience, Roseville, USA). 2×Color SYBR Green qPCR Master Mix (for mRNA) and EZ-Probe qPCR Master Mix for microRNA (EZBioscience, Roseville, USA) were used for subsequent qRT-PCR amplification on ABI Quant-Studio 5 system. Glyceraldehyde-3-phosphate dehydrogenase (GAPDH) and U6 were used as internal controls. The sequences of the gene-specific primers are listed in Table 1. Universal 3'qPCR primer is included in EZ-Probe qPCR Master Mix for microRNA (EZBioscience, Roseville, USA).

**Table 1:** Primer sequences for quantitative reverse-transcription polymerase chain reaction

Gene	Sequence 5′ → 3′
SMAD6	Forward: AGACGGCGTTGGCCTTT
	Reverse: CCTGCCTTTACCTTGCCTTTT
LOC100302640	Forward: GCGGAAGGGGCTTGTTCATT
	Reverse: TGCGTAGGTCAAGTATCGGC
miR-1469	Forward: CTCGGTGCGGGGCG
U6	Forward: CCTGCTTCGGCAGCACA
GAPDH	Forward: AACGGATTTGGTCGTATTGGG
	Reverse: CCTGGAAGATGGTGATGGGAT

Abbreviations: GAPDH, Glyceraldehyde-3-phosphate dehydrogenas; SMAD6, Mothers against decapentaplegic homolog 6.

### Statistical analysis

The data were analysed with SPSS 17.0 software (SPSS, Inc., Chicago, IL, USA). The mean ± standard deviation (mean ± SD) is presented for the quantitative data. Student’s t-test was performed for normally distributed data to determine statistical significance. The level of significance was set at *P*-value < 0.05.

## Results

### Raw data

The lncRNA and mRNA expression data (GSE92681) and miRNA expression data (GSE92681) during the osteogenic differentiation of human PDLSCs were obtained from our two previous studies. In this process, 994 lncRNAs had upregulated expression and 1,177 had downregulated expression (fold change >2.0 or <-2.0; *P* <0.05). A total of 1,578 mRNAs had upregulated expression, and 1,979 mRNAs had downregulated expression. Thirty miRNAs had upregulated expression and 86 had downregulated expression.

### LncRNA–miRNA–mRNA networks

**Fig. 2:**
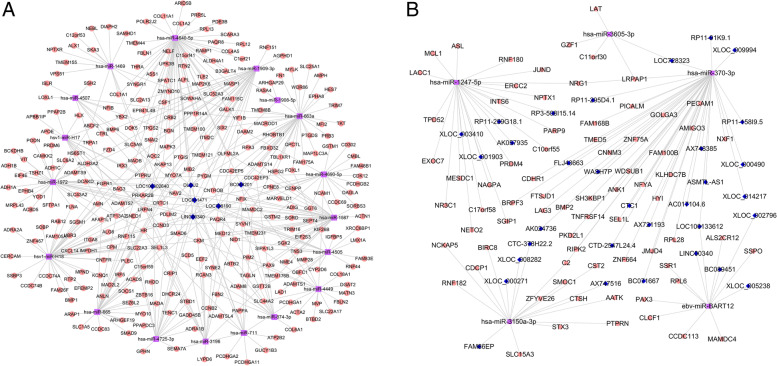
ceRNA network. A: Consisting of lncRNAs with upregulated expression, miRNAs with downregulated expression and mRNAs with upregulated expression; B: Consisting of lncRNAs with downregulated expression, miRNAs with upregulated expression and mRNAs with downregulated expression. The blue diamonds represent lncRNAs, squares coloured purple indicate miRNAs and pink circles are mRNAs

**Fig. 3:**
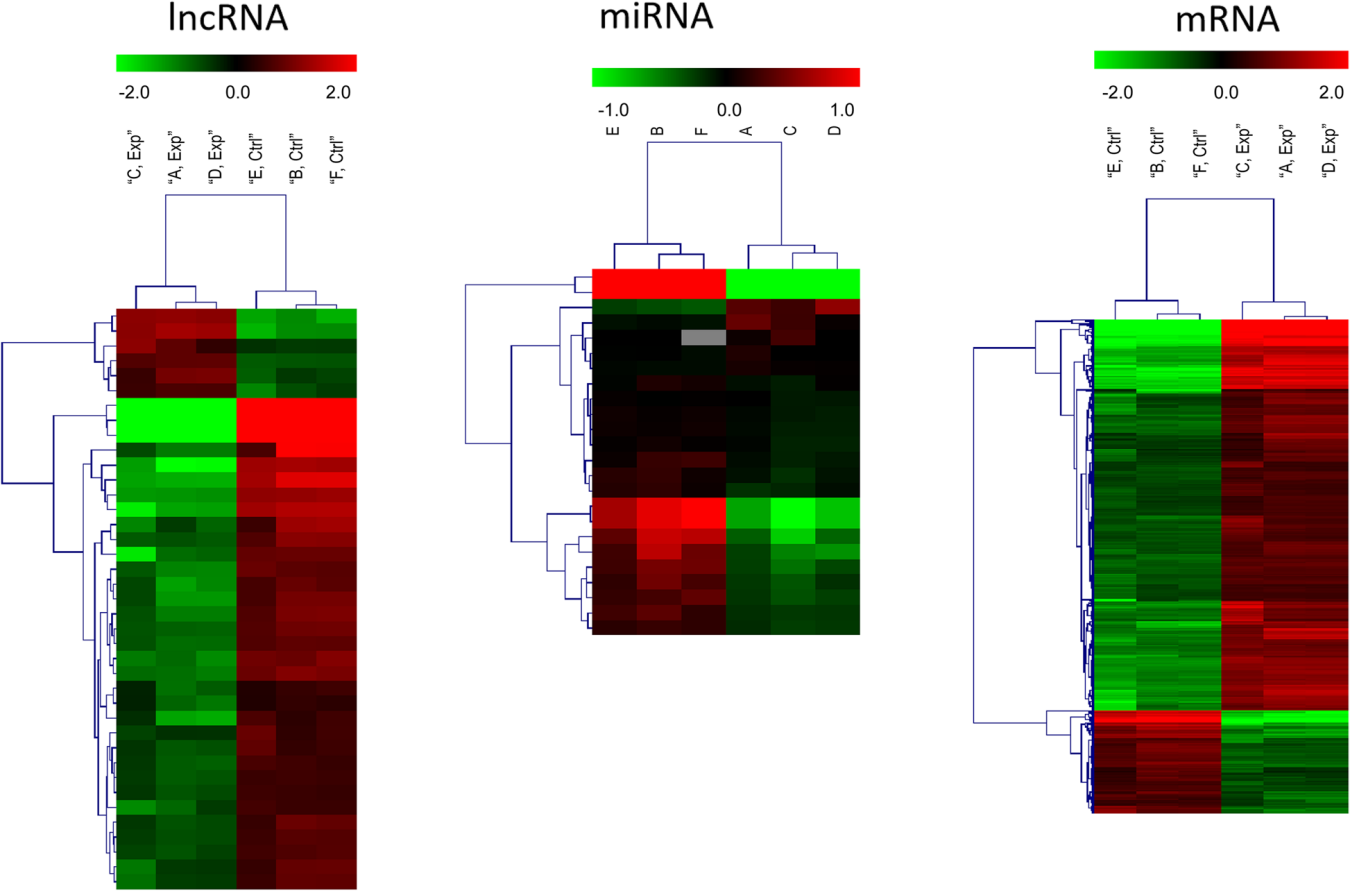
Heatmap of differentially expressed RNAs involved in the ceRNA network. A: Differentially expressed lncRNAs; B: Differentially expressed miRNAs; C: Differentially expressed mRNAs. The expression value is indicated by the colour scale. The intensity increased from blue to red. Each column represents one sample, and each row represents one transcript

For analysing the functions of lncRNAs acting as ceRNAs, a lncRNA–miRNA–mRNA network was first established. As shown in Fig. 2, the URL–DRM–URM network had 6 lncRNA nodes, 280 mRNA nodes and 18 miRNA nodes (Fig. 2A). The DRL–URM–DRM network contained 33 lncRNA nodes, 73 mRNA nodes, and 5 miRNA nodes (Fig. 2B). Heatmaps of lncRNA, miRNA and mRNA expression patterns involved in ceRNA networks are shown in Fig. 3.

### Predicted function of lncRNAs based on the network

**Fig. 4:**
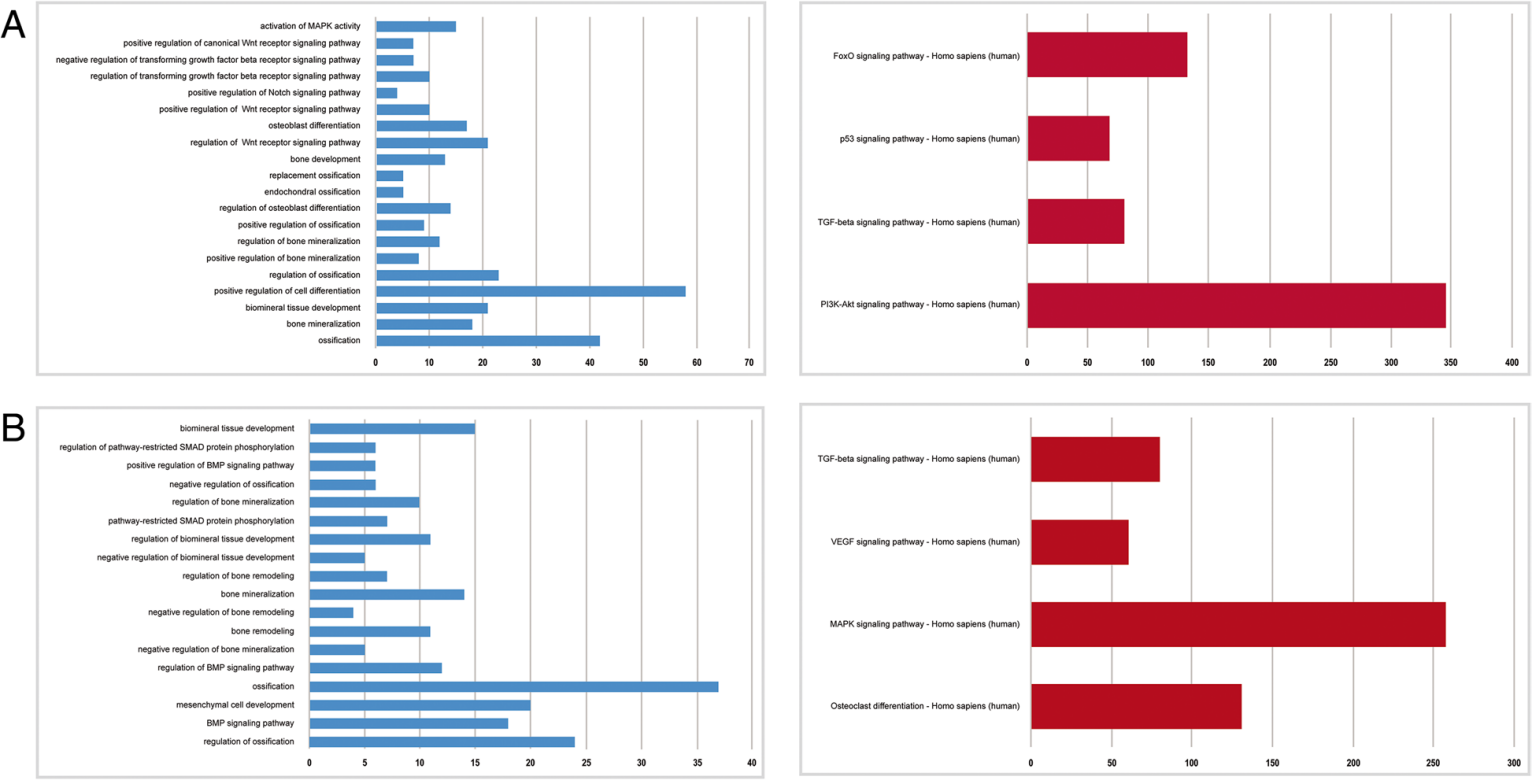
GO annotations for biological processes and KEGG pathway analysis of mRNAs related to osteogenic differentiation. A: Upregulated expression; B: Downregulated expression

The function of each lncRNA could be inferred from the function of the surrounding mRNAs. The mRNAs with upregulated and downregulated expression in the URL–DRM–URM and DRL–URM–DRM networks were included to analyse their functions. The potential regulatory roles of the ceRNA networks were predicted by analysing the functions of 280 mRNAs with upregulated expression and 73 with downregulated expression through GO and KEGG pathway analysis. GO annotations (*P* < 0.05) involving multiple biological processes, cellular components and molecular functions and the top 30 pathways are displayed in Fig. 4. Among these KEGG pathways, the MAPK pathway and TGF-beta regulating pluripotency of stem cells were closely related to osteogenesis.

**Fig. 5:**
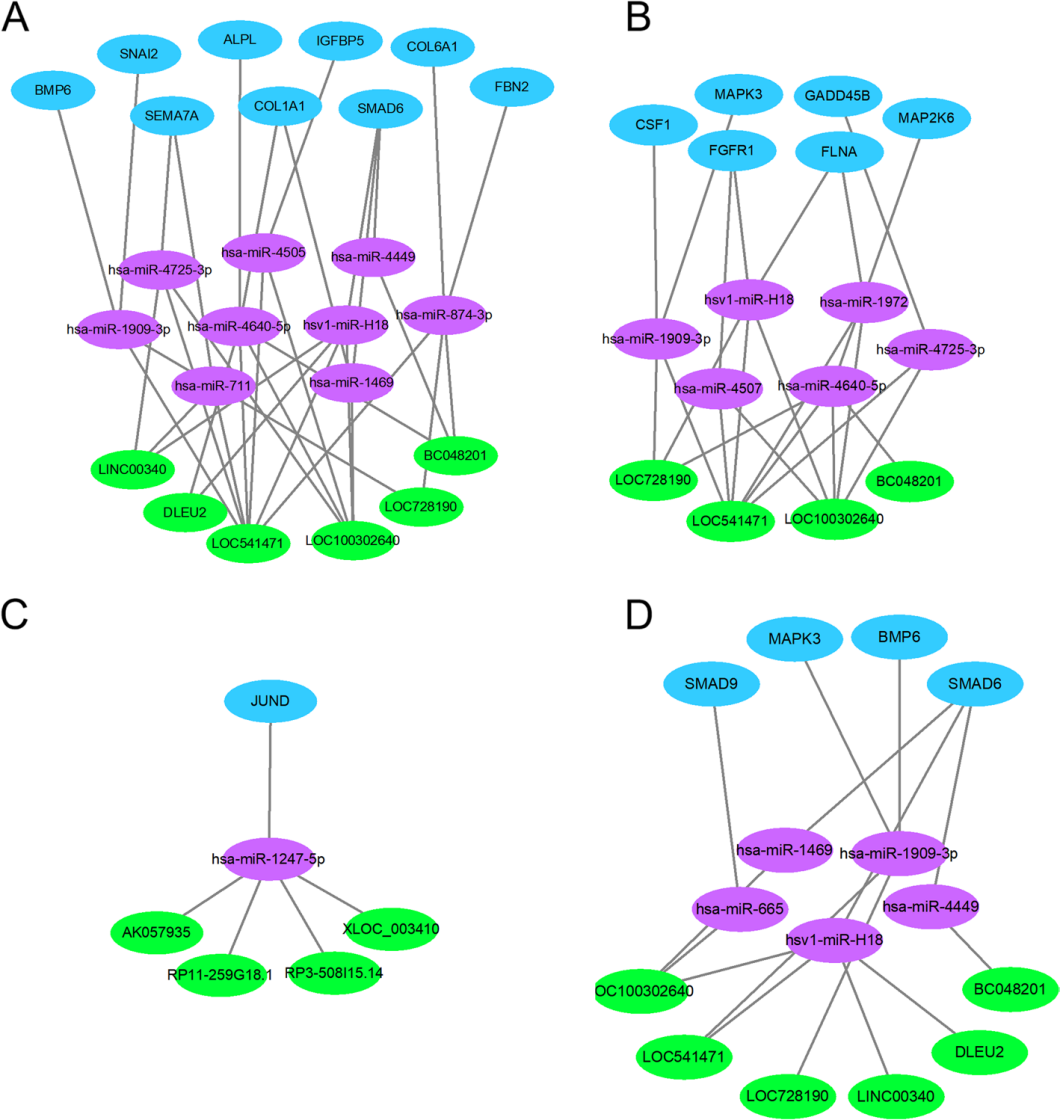
ceRNA networks of lncRNAs-miRNAs-mRNAs. **A**: Significantly participated in GO: 0001649 (osteoblast differentiation); B, C: The mRNAs/lncRNAs with upregulated expression (**B**) and with downregulated expression (**C**) related to the MAPK pathway; D: Significantly participated in TGF-beta pathway

Among these GO terms, we obtained GO: 0001649 (osteoblast differentiation), which was significantly enriched by 9 mRNAs (SMAD6, ALPL, COL6A1, SEMA7A, COL1A1, BMP6, SNAI2, FBN2 and IGFBP5). The complex mRNA networks involved in GO:0001649 (osteoblast differentiation) and 9 related miRNAs and 6 lncRNAs are displayed in Fig. 5A.

Based on the above results, we selected several lncRNAs, miRNAs and mRNAs associated with the MAPK and TGF-beta pathways to further display the ceRNA networks (Fig. 5B-D). This complicated ceRNA network suggested that 8 lncRNAs might play regulatory roles in the MAPK pathway through 7 miRNAs and their targets during the osteogenic differentiation of PDLSCs. Six lncRNAs, 5 miRNAs and their targets in the TGF-beta pathway are involved in this process.

### Validation

**Fig. 6:**
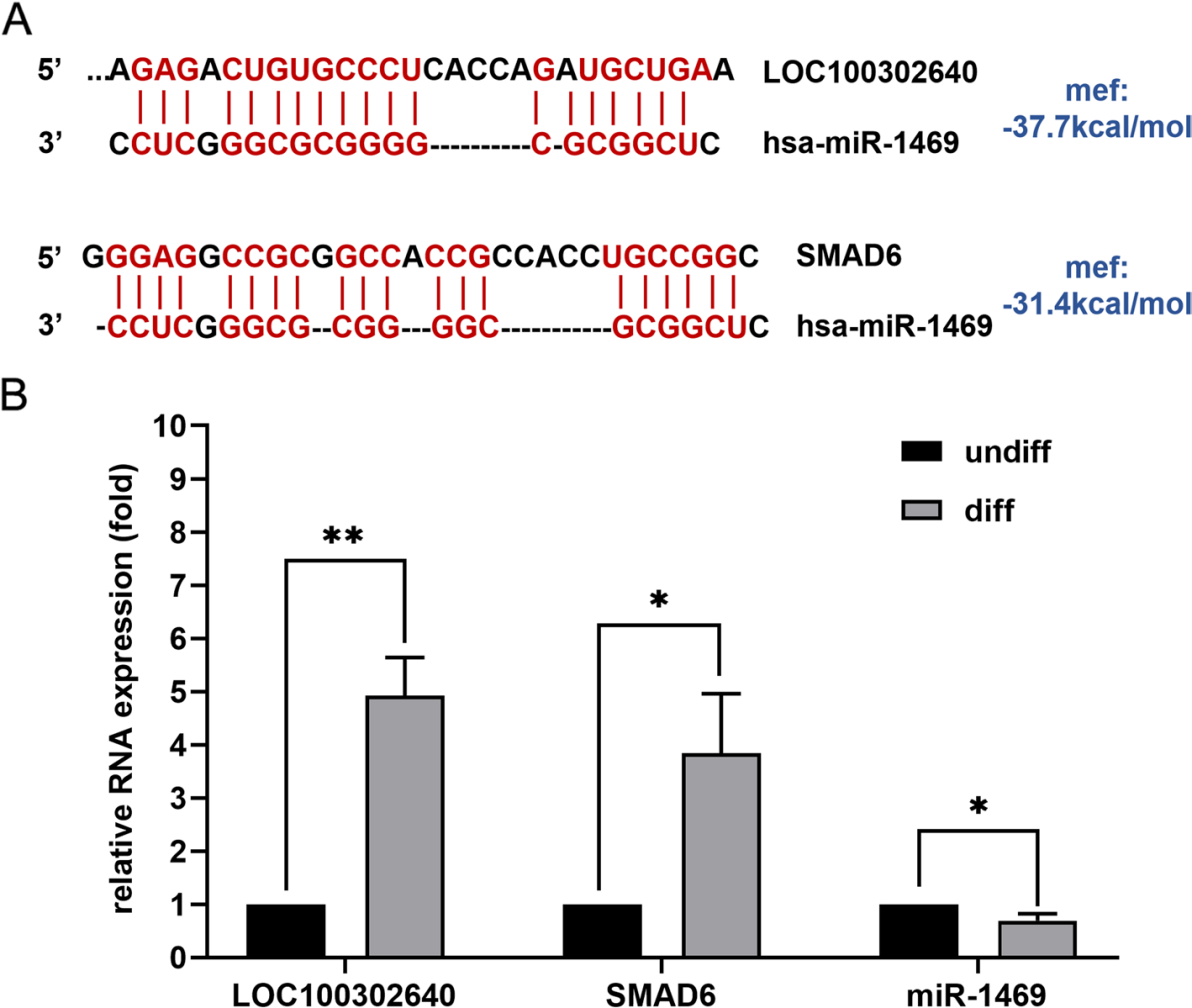
Validation of the key lncRNA–miRNA–mRNA subnetwork of interest. A. The predicted binding sites of LOC100302640/miR-1469 and miR-1469/SMAD6; B. The expression levels of LOC100302640/miR-1469/ SMAD6; SMAD6, Mothers against decapentaplegic homologue 6

Differentially expressed RNAs of interest were selected. The binding sites of LOC1001302640/miR-1469 and miR-1469/SMAD6 were predicted as shown in Fig. 6A, and the expression levels of them were validated by qRT-PCR. The validation results were consistent with the microarray analysis data. LOC1001302640 and SMAD6, which had upregulated expression in the differentiation group, showed significantly higher levels than those in the undifferentiation group. Downregulation of miR-1469 expression in the differentiation group resulted in significantly lower levels than that in the undifferentiation group (Fig. 6B).

### Discussion

Comprehensive analysis concerning the ceRNA network has been established in ankylosing spondylitis [28], rheumatoid arthritis [29], cervical cancer [30], and cutaneous melanoma [31], periodontitis [32, 33], as well as tissue regeneration involing dental pulp stem cell [9, 34]. Over 280 papers reported the osteogenic ability of PDLSCs. As a potential candidate for tissue regeneration, PDLSCs worth certain attentions. Besides, ceRNA mechanism has been reported to play a significant role in osteogenesis of PDLSCs [35, 36], however, a comprehensive analysis is still wanting. Thus, it is needed to provide a view of PDLSCs-related analysis for researchers who are interested in PDLSCs. Existed papers were based on microarrays of lncRNA and mRNA and bioinformatic prediction of miRNA [35, 36], instead, the two databases we used that report lncRNA and miRNA respectively [19, 20] is derived from the same samples, which ensures the consistence and comparability of the analysis. Based on these two datasets, we successfully constructed a ceRNA network through bioinformatic analyses.

Among the 6 lncRNAs with upregulated expression in the ceRNA network, 4 were not reported (BC048201, LOC100302640, LOC728190, LINC00340), among which LOC100302640 had the highest expression level in the microarray data, and it was predicted that it may be related to the MAPK and TGF-beta pathways. In addition, LOC541471 was identified as an overall survivor-related lncRNA in glioblastoma multiforme [37]. Another study reported that LOC541471 is a novel prognostic biomarker for head and neck squamous cell carcinoma [38]. The leukaemia 2 gene (DLEU2) has been reported to play an important role in a variety of diseases [39, 40], especially in tumour diseases, including thyroid cancer, gastric cancer, sarcopenia, and laryngeal squamous cell carcinoma, and has been shown to play a role through the ceRNA mechanism [41–44].

In addition to these lncRNAs revealed in these paper, some classical lncRNAs, such as SNHG1, TUG1, GAS5, XIST, DANCR and FER1L4, were reported to be involved in the osteogenic differentiation of PDLSCs [17, 45–49], most of them function as ceRNAs (miRNA sponges) to regulate osteogenesis. In order to reveal a lncRNA-miRNA-mRNA network, a complete study includes procedures as follows. From RNA-sequencing data, we find out the differentially expressed genes at first. Secondly, based on the reliable database that involved in the field of interest, the enriched mRNAs were screened out by GO analysis. With predicted softwares, such as miRanda, we obtain related lncRNA and miRNA. Together with the mRNA, they compose the network. Furthermore, qRT-PCR, lentivirus transfection, rescue, pull-down, and luciferase assays are needed to be carried out to verify the network.

GO and pathway analyses were carried out to explore biological functions enriched among mRNAs with upregulated and downregulated expression. GO analysis was used as a controlled repertoire to investigate the function of mRNAs with upregulated and downregulated expression and describe mRNA and mRNA products that are distributed in any organism. Pathway analysis was conducted to identify genes with upregulated and downregulated expression based on KEGG analysis. In our research, the results of GO analysis showed that 678 GO enriched terms were significant with *P*-value < 0.05 in the mRNAs with upregulated expression. These significant GO terms involved the transforming growth factor beta receptor signalling pathway, regulation of Wnt receptor signalling pathway, positive regulation of Notch signalling pathway, enzyme-linked receptor protein signalling pathway. In the mRNAs with downregulated expression, we found 714 GO enriched terms that were significant with a *P*-value < 0.05. These significant GO terms involved the BMP signalling pathway, Toll signalling pathway, mesenchymal cell differentiation, and regulation of cell differentiation. The most significant pathways, such as the TGF-beta signalling pathway, MAPK signalling pathway, p53 signalling pathway, and FoxO signalling pathway, are related to osteogenesis. We further predicted the lncRNAs involved in the GO terms 0001649 (osteoblast differentiation), MAPK and TGF-β and found that LOC100302640, which had upregulated expression in the osteogenic differentiation, suggesting that this new LOC100302640 may participate in the regulation of PDLSC osteogenesis through a ceRNA mechanism. According to ceRNA theory, LOC100302640 may act as a ceRNA, competitively bind with miR-1469, which releases the suppression of SMAD6 by miR1469 to promote the osteogenesis of PDLSCs, However, the specific mechanism of this ceRNA network requires further verification.

## Conclusion

The present novel findings on the differential expression of lncRNAs, miRNAs and mRNAs in the osteogenic differentiation of human PDLSCs suggest the essential involvement of ncRNAs and related regulatory mechanisms in PDLSC osteogenesis. LOC100302640 had the most significantly upregulated expression, which provided further support for our prediction of the lncRNA-miRNA-mRNA ceRNA network. Each ceRNA pair identified might be a potential candidate regulator of PDLSC osteogenesis, and further experiments are needed to explore the specific underlying genetic traits and interaction networks.

## Supplementary Information

**Additional file 1.**

**Additional file 2.**

**Additional file 3.**

**Additional file 4.**

**Additional file 5.**

**Additional file 6.**

**Additional file 7.**

## Data Availability

The data of microarrays are available in GEO database. (GSE159507 and GSE159508)

## Abbreviations

PDLSCs: periodontal ligament stem cells
LncRNAs: Long noncoding RNAs
mRNA: Messenger RNA
ncRNAs: Noncoding RNAs
miRNA: microRNA
mRNA: messenger RNA
CeRNA: Competitive endogenous RNA
GO: Gene Ontology
DAVID: Database for Annotation, Visualization, and Integration Discovery
KEGG: Kyoto Encyclopedia of Genes and Genomes
RT-PCR: Quantitative reverse-transcription polymerase chain reaction
URLs: lncRNAs with upregulated expression
DRMis: miRNAs with downregulated expression
URMs: mRNAs with upregulated expression
DRLs: lncRNAs with downregulated expression
URMis: miRNAs with upregulated expression
DRMs: mRNAs with downregulated expression
GAPDH: Glyceraldehyde-3-phosphate dehydrogenase
MAPK: Mitogen-activated protein kinase
