# Supplementary material for: Comprehensive analysis of the long noncoding RNA-associated competitive endogenous RNA network in the osteogenic differentiation of periodontal ligament stem cells
Source: BMC Genomics. 2022 Jan 3;23:1. doi: 10.1186/s12864-021-08243-4 (PMC8725252; doi:10.1186/s12864-021-08243-4)
Supplement: Supplementary file 1 — Additional file 1. [file 12864_2021_8243_MOESM1_ESM.doc]

**SUPPLEMENTAL MATERIALS AND METHODS**

**ALP and ARS staning assay**

Alkaline phosphatase (ALP) staining assay and alizarin red staining (ARS) were used to confirm the osteogenic induction model. On the 7 day of osteogenic induction, uninduced and induced PDLSCs was stained with Alkaline phosphatase assay kit (Nanjing Jiancheng Bioengineering Institute, A059). On the 14 day of induction, the cells were respectively stained with alizarin red (Cyagen, HUXMA-90021).
