# Supplementary material for: Comprehensive analysis of the long noncoding RNA-associated competitive endogenous RNA network in the osteogenic differentiation of periodontal ligament stem cells
Source: BMC Genomics. 2022 Jan 3;23:1. doi: 10.1186/s12864-021-08243-4 (PMC8725252; doi:10.1186/s12864-021-08243-4)
Supplement: Supplementary file 2 — Additional file 2. [file 12864_2021_8243_MOESM2_ESM.docx]

***Figure S1.*** **Osteogenic differentiation of hPDLSCs.** Alkaline phosphatase staining in undifferentiation group (A) and differentiation group (B) (×100). Alizarin Red staining was performed to detect mineral nodes in undifferentiation group (C) and differentiation group (D) (×100).
