# Supplementary figures and images for: Comprehensive analysis of the long noncoding RNA-associated competitive endogenous RNA network in the osteogenic differentiation of periodontal ligament stem cells
Source: BMC Genomics. 2022 Jan 3;23:1. doi: 10.1186/s12864-021-08243-4 (PMC8725252; doi:10.1186/s12864-021-08243-4)

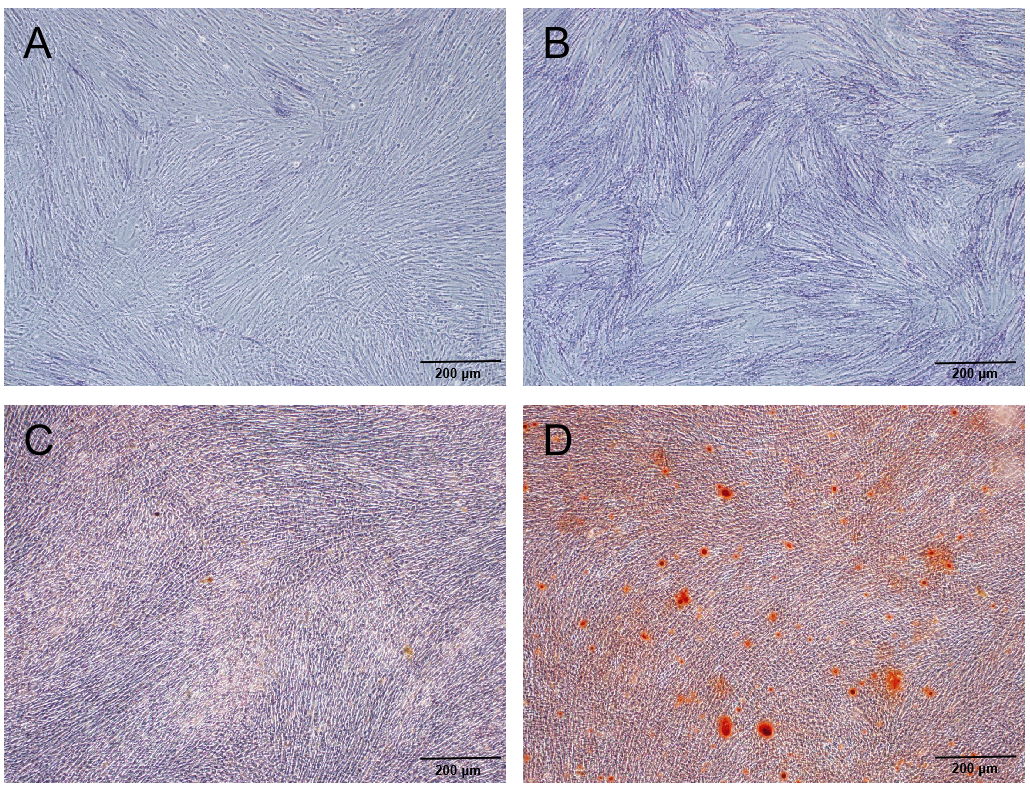

Supplement: Supplementary file 3 — Additional file 3. [file 12864_2021_8243_MOESM3_ESM.tif]
