# Supplementary material for: Comprehensive analysis of the long noncoding RNA-associated competitive endogenous RNA network in the osteogenic differentiation of periodontal ligament stem cells
Source: BMC Genomics. 2022 Jan 3;23:1. doi: 10.1186/s12864-021-08243-4 (PMC8725252; doi:10.1186/s12864-021-08243-4)
Supplement: Supplementary file 7 — Additional file 7. [file 12864_2021_8243_MOESM7_ESM.docx]

**Table S4 Basic information of tooth donors**

| Number | Age | Sex | Tooth position |
| --- | --- | --- | --- |
| 1 | 18 | Female | 18/28 |
| 4 | 22 | Male | 38/48 |
| 5 | 20 | Male | 18/28 |
| 6 | 24 | Female | 18/28 |
| 7 | 22 | Male | 38/48 |
| 8 | 20 | Male | 18/28 |
| 9 | 23 | Female | 18/28 |
| 10 | 24 | Female | 18/48 |
| 11 | 18 | Male | 18/28 |
| 12 | 18 | Male | 18/48 |
| 13 | 21 | Female | 18/48 |
